# Mesenchymal Stem Cell Enhances the Function of MDSCs in Experimental Sjögren Syndrome

**DOI:** 10.3389/fimmu.2020.604607

**Published:** 2020-12-22

**Authors:** Jie Tian, Yue Hong, Qiugang Zhu, Huimin Zhou, Yidan Zhang, Ziwei Shen, Hongye Guo, Yue Zhang, Xiangyan Ai, Futao Zhao, Ke Rui, Huaxi Xu, Shengjun Wang

**Affiliations:** ^1^ Department of Laboratory Medicine, The Affiliated People’s Hospital, Jiangsu University, Zhenjiang, China; ^2^ Department of Immunology, Jiangsu Key Laboratory of Laboratory Medicine, School of Medicine, Jiangsu University, Zhenjiang, China; ^3^ Department of Rheumatology, Shanghai Ninth People’s Hospital, Shanghai Jiao Tong University School of Medicine, Shanghai, China; ^4^ Department of Laboratory Medicine, Affiliated Hospital of Jiangsu University, Zhenjiang, China

**Keywords:** bone marrow-mesenchymal stem cell, myeloid-derived suppressor cell, Sjögren’s syndrome, TGF-β, autoimmune disease

## Abstract

Primary Sjögren’s syndrome (pSS) is a progressive systemic autoimmune disease characterized by lymphocytic infiltrates in exocrine glands, leading to the injury of salivary and lachrymal glands. Mesenchymal stem cells (MSCs) have been demonstrated to exert great potential in the treatment of various autoimmune diseases. Although MSCs have provide an effective therapeutic approach for SS treatment, the underlying mechanisms are still elusive. Our previous study has shown the reduced suppressive capacity of myeloid-derived suppressor cells (MDSCs) advanced the progression of experimental Sjögren’s syndrome (ESS). In this study, we found that BM-MSCs significantly enhanced the suppressive function of MDSCs with high levels of Arginase and NO, decreased the levels of CD40, CD80, CD86, and MHC-II expression on MDSCs, thus attenuating the disease progression in ESS mice. Furthermore, the enhanced suppressive function of MDSCs was mediated by BM-MSC-secreted TGF-β, and the therapeutic effect of BM-MSCs in inhibiting ESS was almost abolished after silencing TGF-β in BM-MSCs. Taken together, our results demonstrated that BM-MSCs alleviated the ESS progression by up-regulating the immunosuppressive effect of MDSCs through TGF-β/Smad pathway, offering a novel mechanism for MSCs in the treatment of pSS.

## Introduction

Primary Sjögren’s syndrome (pSS) is a chronic, systemic autoimmune disease characterized by lymphocytic infiltrates in salivary and lacrimal glands, leading to the destruction of these exocrine glands. The common symptoms are xerostomia and xerophthalmia (1). Besides the characteristic glandular symptoms, other systemic extraglandular manifestations, including synovitis, interstitial lung disease, vasculitis and renal diseases (2). Moreover, approximately 5% of patients with pSS may develop lymphoma, mainly the mucosa-associated lymphoid tissue non-Hodgkin lymphoma, which is the most severe complication of the disease (3). pSS is considered to be essentially driven by a complex interaction between epithelial barrier and adaptive and innate immunity. Macrophages, dendritic cells, NK cells, T cells (Th1, Th2, Th17, Tfh, Tfr, Treg), and B cells have been reported to be involved in the pathogenesis of the disease (3–6). Additionally, our previous work has clarified the essential role of MDSCs in the progression of pSS (7). Currently, treatment of Sjögren’s syndrome patients is still challenging due to the complex pathogenesis of the disease, approaches such as biologic agents and traditional disease-modifying antirheumatic drugs cannot cure this disease and have some side effects (8). Therefore, exploring novel therapeutic approaches is critically necessary for the treatment of pSS.

Myeloid-derived suppressor cells (MDSCs) are a heterogeneous population of immature myeloid cells, which has emerged as a universal regulator of immune function under many pathologic conditions (9). MDSCs can be subdivided into two major subpopulations: polymorphonuclear MDSCs (PMN-MDSCs) with a CD11b^+^Ly-6G^+^Ly-6C^lo^ phenotype and monocytic MDSCs (M-MDSCs) with a CD11b^+^Ly-6G^-^Ly-6C^hi^ phenotype, both are characterized by the expression of CD11b^+^Gr-1^+^. PMN-MDSCs exert their suppressive effect mainly by high levels of arginase 1 and reactive oxygen species (ROS) whereas M-MDSCs produce NO. In healthy individuals, MDSCs are generated in bone marrow and quickly differentiate into mature dendritic cells, macrophages, or granulocytes. However, under pathological conditions, the differentiation of MDSCs will be blocked and cause the expansion of this population *in vivo* (9). Recently, MDSCs have been demonstrated to be involved in the pathogenesis of various autoimmune diseases, including rheumatoid arthritis (RA), systemic lupus erythematosus (SLE), type 1 diabetes and multiple sclerosis (MS) (10–15). Our previous studies have characterized a pivotal role of MDSCs in the development of SS. We found that MDSCs were significantly increased in mice with experimental Sjögren’s syndrome (ESS), but their suppressive function of MDSCs was gradually decreased with the progression of the disease, and eventually leading to the uncontrollable inflammatory responses and irreversible tissue injury. Therefore, restoring or enhancing the suppressive capacity of MDSCs are supposed to be a promising therapeutic strategy for pSS.

Mesenchymal stem cells (MSCs) is a group of mesodermal and ectodermal origin multipotent stromal cells with the capacity of self-renewal and differentiation into osteoblasts, adipocytes, and chondrocytes (16). The properties of rapid proliferation and powerful immunomodulation have entitled their potential application in the treatment of various debilitating diseases (17). Indeed, MSCs have been reported to exert immunomodulatory effects on T cells, B cells, dendritic cells, and natural killer cells (18), which makes them a promising therapy for various autoimmune diseases, including systemic lupus erythematosus (19, 20), rheumatoid arthritis (21), inflammatory bowel disease (22) and systemic sclerosis (23). However, much less is known about the effects of MSCs in treating Sjögren’s syndrome, and the underlying mechanism still remains to be elucidated.

In this study, we characterized the effect of BM-MSCs on the suppressive capacity of MDSCs in ESS mice, and clarified the regulation was mainly mediated by TGF-β/Smad pathway. Our study offers new insights into the mechanisms of the application of MSCs as a therapy for pSS.

## Methods and Materials

### Mice

Female C57BL/6 mice at 8-week-old and male C57BL/6 mice at 6-week-old were purchased from Experimental Animal Center of Yangzhou University. Mice were housed in a specific pathogen-free animal facility and all the experiments were approved by the Institutional Committee on the Use of Animals for Research and Teaching.

### Induction of ESS Model

The ESS mouse model was induced as previously described (7). Briefly, bilateral salivary glands were isolated from female C57BL/6 mice (8-week-old) for homogenization in PBS to prepare SG proteins. Naïve mice were immunized with SG proteins emulsified in an equal volume of CFA (Sigma-Aldrich) to a concentration of 2 mg/ml (100 µl/mouse) s.c. on the neck on days 0 and 7. On day 14, the booster injection was performed with a dose of 1 mg/ml SG proteins emulsified in Freund’s incomplete adjuvant (Sigma-Aldrich).

### Detection of Saliva Flow Rate

Saliva flow rates were measured as previously described (7). Briefly, mice were anesthetized and injected intraperitoneally with pilocarpine (Sigma-Aldrich) at a dose of 5 mg/kg body weight. Saliva was then collected using a 20-μl pipet tip from the oral cavity for 15 min.

### Autoantibody and Cytokine Detection

Autoantibodies against SG proteins and anti-M3 muscarinic receptor (M3R) antibodies and ANA (Elabscience) were measured with a sandwich enzyme-linked immunosorbent assay (ELISA) as previously described (7). Briefly, 96-well plates were pre-coated with the antigen at 4°C overnight. Samples were incubated for 2 h at room temperature, followed by incubation of biotin-conjugated anti-mouse IgG for 1 h. After washing, HRP Streptavidin was added and incubated for 30 min. Then, plates were washed and the TMB substrate was added. After 30 min, stop solution was added and absorbance was measured at 450 nm using a microplate reader (BioTek, Winooski). Mouse serum levels of IL-17 and IFN-γ were measured with ELISA Kits (eBioscience) following the manufacturer’s protocol.

### Isolation and Culture of BM-MSCs

For the culture of BM-MSCs, bone marrow (BM) cells were isolated from C57BL/6 mice (6-week-old) and cultured in the medium (DMEM supplemented with 15% fetal calf serum) (Gibco) for 3 days. Non-adherent cells were then removed and when the remaining cells reached 80% confluence in the dish, the adherent cells were expanded for three passages and used for the subsequent experiments.

### MDSC Isolation

CD11b^+^Gr-1^+^ MDSCs were isolated from the spleens of ESS mice using a FACSAria II SORP (Becton Dickinson) cell sorter (Miltenyi Biotec). M-MDSCs and PMN-MDSCs were isolated using a mouse MDSC isolation kit (Miltenyi Biotec) following the manufacturer’s protocol.

### Flow Cytometric Analysis

For surface markers, single-cell suspensions were stained with relevant fluorochrome-conjugated monoclonal antibodies(mAbs): anti-mouse CD40 (HM40-3), CD80 (16-10A1), CD86 (GL1), and MHCII (M5/114.15.2) from eBioscience, anti-mouse CD11b (M1/70), Gr-1 (RB6-8C5), Ly6G (1A8), and Ly6C (HK1.4) from Biolegend, For intracellular staining, cells were stimulated with PMA (Sigma-Aldrich, 50 ng/ml), ionomycin (Enzo, 1 µg/ml), monensin (Enzo, 2 µg/ml). After 5 h, cells were stained with antibodies against surface markers, fixed, permeabilized, and stained with anti-IFN-γ mAb (XMG1.2, eBioscience), anti- IL-17 mAb (eBio17B7, eBioscience), or anti-TGF-β mAb (TW7-16B4, eBioscience) according to the Intracellular Staining Kit (Invitrogen) instructions. Flow cytometry was performed using the BD FACSCanto II (Becton Dickinson) and data were analyzed using FlowJo software (Treestar).

### Quantitative Real-Time PCR

The quantitative real-time PCR were performed as previously described. The sequences for the primers used are: TGF-β, Forward -5’- AACCGGCCCTTCCTGCTCCTCAT -3’, Reverse-5’- CGCCCGGGTTGTGTTGGTTGTAGA -3’. β-actin, Forward -5’-TGGAATCCTGTGGCATCCATGAAAC-3’, Reverse-5’-TAAAACGCAGCTCAGTAACAGTCCG-3’. β-actin was used as an internal control.

### T Cell Suppression Assay

Mouse CD4^+^ T cells were sorted from wild-type mice using CD4^+^T cell microbeads (Miltenyi Biotec). CD4^+^ T cells were labeled with carboxyfluorescein succinimidyl ester (CFSE, 5 mM; Invitrogen), and then co-cultured with MDSCs at a ratio of 1:1 in 96-well plates (Costar) in the presence of anti-CD3 and anti-CD28 mAbs (eBioscience) for 3 days. CFSE fluorescence intensity was analyzed to determine the proliferation of CD4^+^ T cells by flow cytometry.

### Western Blot

Proteins extracted from cells were prepared as previously described. Equal amounts of proteins were separated by 12% SDS-PAGE, then transferred onto Immobilon polyvinylidene difluoride membranes (Bio-Rad). Antibodies against Smad2/3 and p-Smad2/3 were purchased from Cell Signaling Technology.

### Histologic Analysis

After mice were euthanatized, submandibular glands were collected and immediately fixed in 4% paraformaldehyde. Paraformaldehyde-fixed tissues were embedded in paraffin. Serial 4-μm sections were cut and stained with hematoxylin and eosin (H&E) for morphologic examination. The severity of the tissue damage was evaluated using the following scoring system. A lymphocytic focus was defined as a group of ≥50 lymphocytes. The focus score (FS) was classified as: FS=0: no lymphocytic infiltration; FS=1: <1 lymphocytic focus per 4 mm^2^ (0<FS<1); FS=2: <2 lymphocytic foci per 4 mm^2^; FS=3: two or more lymphocytic foci per 4 mm^2^ (24).

### Detection of Arginase Activity and NO Production

The activity of arginase and NO concentration were measured as previously described (25). The arginase activity was determined for the conversion of arginine to ornithine and urea by a quantitative colorimetric assay employing a QuantiChrom arginase assay kit (BioAssay Systems). The arginase activity was calculated according to the manufacturer’s instructions. The amount of NO was assessed by determining the concentration of nitrite accumulated in culture supernatants using the colorimetric Griess reaction (Promega).

### Transfection

TGF-β siRNA and the negative control were synthesized by RiboBio. Oligonucleotide transfection was performed with Entranster-R (Engreen Biosystem) according to the manufacturer’s instructions.

### Statistical Analysis

The statistical significance was determined by the Student’s t test or one-way ANOVA. All analyses were performed using SPSS 16.0 software. p Values less than 0.05 were considered statistically significant.

## Results

### Adoptive Transfer of BM-MSCs Effectively Alleviates the Progression of ESS

**Figure 1:**
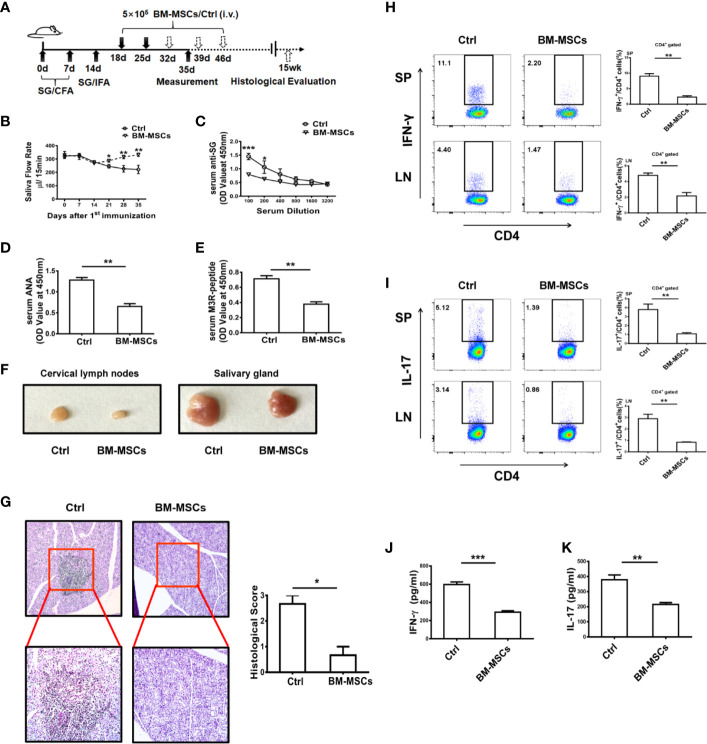
BM-MSCs suppress the progression of ESS. **(A)** Graphic scheme of ESS induction and BM-MSCs administration. C57BL/6 mice were immunized with SG/CFA on days 0 and 7, and mice were boosted with SG/IFA on day 14. Treatment groups were intravenously injected with 5×10^5^ BM-MSCs on days 18 and 25. Mice were sacrificed on day 35 (n=6). **(B)** The saliva flow rates were measured in each group. **(C–E)** Autoantibodies against SG antigens **(C)**, ANA **(D),** and anti-M3R antibodies **(E)** were detected in the serum of ESS mice on day 35. **(F)** Representative graphs show the sizes of CLN and SG. **(G)** ESS mice were transferred with BM-MSCs on days 18, 25, 32, 39 and 46, the histological evaluation of glandular destruction in each group was performed on tissue sections of submandibular glands with H&E staining 15 weeks post first immunization. **(H, I)** Both proportions and numbers of CD4^+^IFN-γ^+^ Th1 cells **(H)** and CD4^+^IL-17^+^ Th17 cells **(I)** were measured in SP and CLN of mice with different treatment on day 35. **(J, K)** Serum levels of IFN-γ and IL-17 were detected in different groups on day 35. Data are shown as mean ± SD of three independent experiments, n=6/group. ***p < 0.001, **p < 0.01, *p < 0.05.

BM-MSCs were adoptively transferred into ESS mice on days 18 and 25, and then the therapeutic effect of the cells in SS was evaluated (**Figure 1A**). Remarkably, BM-MSCs treatment effectively ameliorated the saliva flow rate and reduced the serum autoantibodies against total SG antigens, ANA, and anti-M3R Abs (**Figures 1B–E**). Notably, the BM-MSCs treated group displayed smaller cervical lymph nodes (CLNs) and salivary glands (SG) while compared to the control group (**Figure 1F**). In addition, histological analysis showed only a small amount of lymphocytic infiltration in local SG from ESS mice treated with BM-MSCs (**Figure 1G**). Furthermore, frequencies of Th1 and Th17 cell populations in spleens and CLNs were also decreased after the BM-MSCs treatment (**Figures 1H, I**), and the similar results were observed in the serum IFN-γ and IL-17 (**Figures 1J, K**). Together, BM-MSCs were demonstrated to suppress the development the ESS.

### BM-MSCs Expand MDSCs With Strong Suppressive Function in ESS

**Figure 2:**
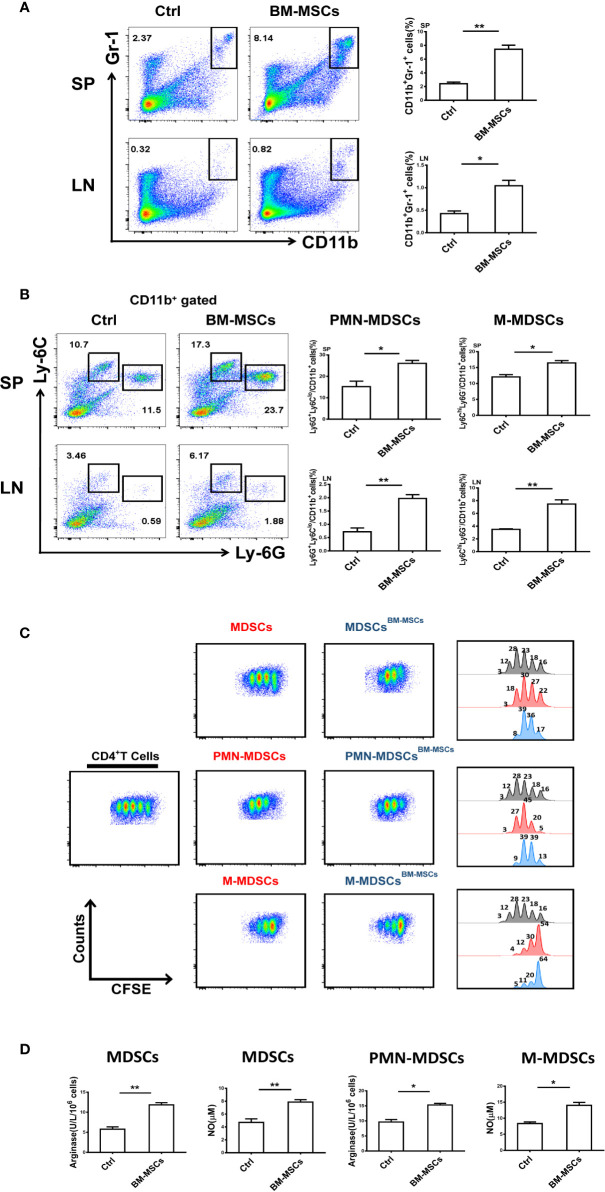
BM-MSCs enhance the suppressive capacity of MDSCs in ESS mice. **(A, B)** Proportions of CD11b^+^Gr-1^+^ MDSCs **(A)**, M-MDSCs and PMN-MDSCs **(B)** were detected in SP and LN after BM-MSCs treatment (n=6). **(C)** Total MDSCs and their subsets from BM-MSCs treated group were isolated, and then co-cultured with CD4^+^T cells in the presence of anti-CD3 and anti-CD28 mAbs for 72 h (MDSC:T cell ratio 1:1). CD4^+^ T cell proliferation was evaluated by staining with CFSE. **(D)** The activity of arginase and the level of NO were measured in MDSCs and their subsets (n=6). Data are shown as means ± SD from three independent experiments, n=6/group. **p < 0.01, *p < 0.05.

Our previous findings have shown that MDSCs in ESS mice gradually lost their suppressive effect during the progression of the disease, which has been determined to be a critical element in the pathogenesis of pSS (7). Therefore, restoring the suppressive capacity of MDSCs in ESS mice might be an efficient strategy for the immunotherapy in pSS. As shown in **Figures 2A, B**, the proportions of MDSCs in spleens and CLNs, including the subsets, PMN-MDSCs and M-MDSCs, were strikingly increased after BM-MSCs treatment. Furthermore, the expanded MDSCs (PMN-MDSCs/M-MDSCs) displayed stronger immunosuppressive effect on T cell proliferation in BM-MSCs treated mice, and expressed higher levels of arginase and NO (**Figures 2C, D**).

### BM-MSCs Enhance the Suppressive Capacity of MDSCs From ESS Mice *In Vitro*

**Figure 3:**
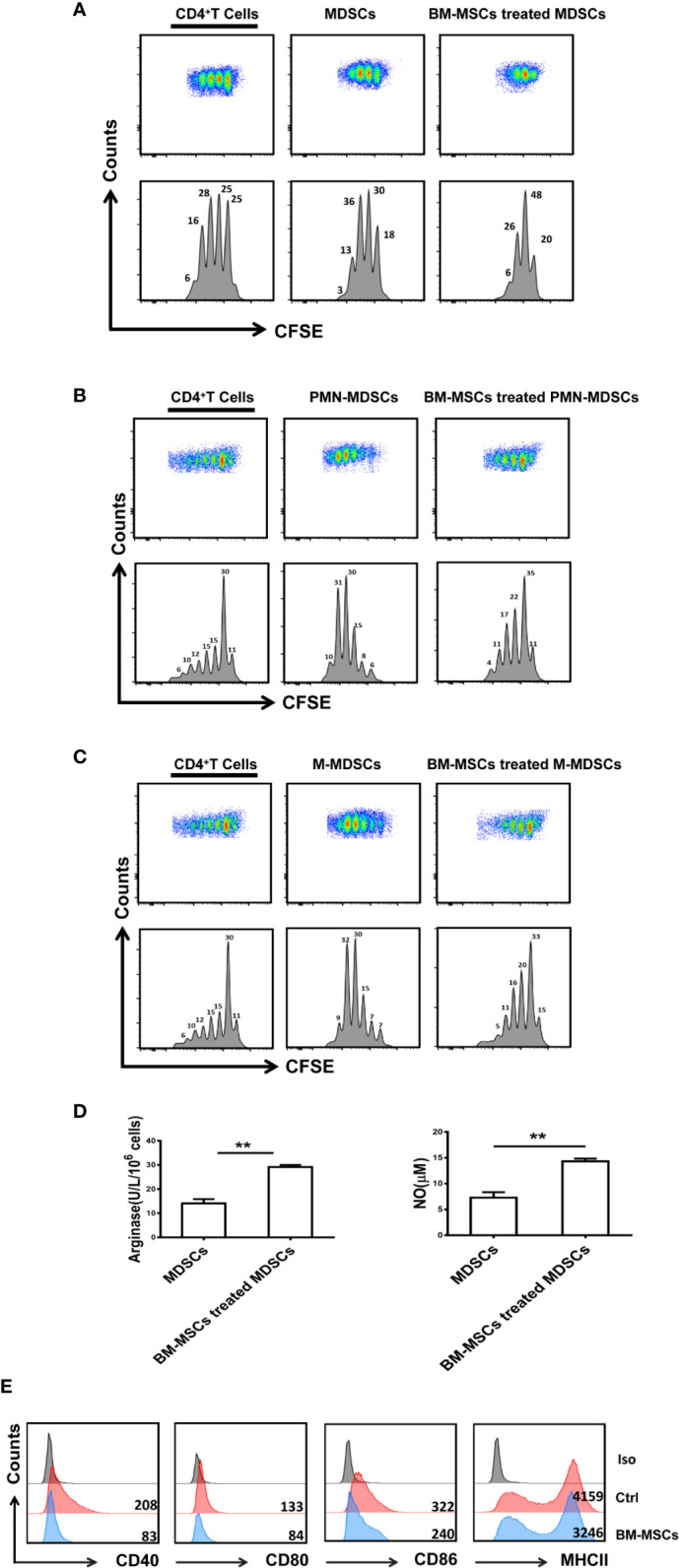
BM-MSCs up-regulate the immunosuppressive function of MDSCs *in vitro*. **(A–C)** Total MDSCs **(A)**, PMN-MDSCs **(B),** and M-MDSCs **(C)** isolated from the spleens of ESS mice were treated with BM-MSCs for 48 h, and then MDSCs were collected for co-culture with CD4^+^T cells in the presence of anti-CD3 and anti-CD28 mAbs for 72 h (MDSC:T cell ratio 1:1). CD4^+^ T cell proliferation was evaluated by staining with CFSE. **(D)** BM-MSCs treated MDSCs were used to measure the activity of arginase activity and the level of NO. **(E)** The expression of CD40, CD80, CD86, and MHCII on MDSCs in two groups was analyzed by flow cytometry. Data are shown as mean ± SD from three independent experiments. **p < 0.01.

The suppressive function of MDSCs (PMN-MDSCs/M-MDSCs) treated with BM-MSCs was measured. MDSCs isolated from ESS mice showed weak suppressive capacity on CD4^+^T cell proliferation. However, after the treatment of BM-MSCs, the suppressive function of MDSCs was enhanced with high levels of arginase and NO, although the suppressive effect of M-MDSCs on CD4^+^ T cell proliferation was only slightly enhanced (**Figures 3A–D**). Additionally, the expression of CD40, CD80, CD86, and MHCII was also down-regulated when compared to the group without BM-MSCs treatment (**Figure 3E**). Together, the *in vitro* experiment further confirmed that BM-MSCs could reverse MDSCs to the immature state with strong suppressive function directly.

### Enhanced Suppressive Function of MDSCs Is Mediated by BM-MSC-Secreted TGF-β

**Figure 4:**
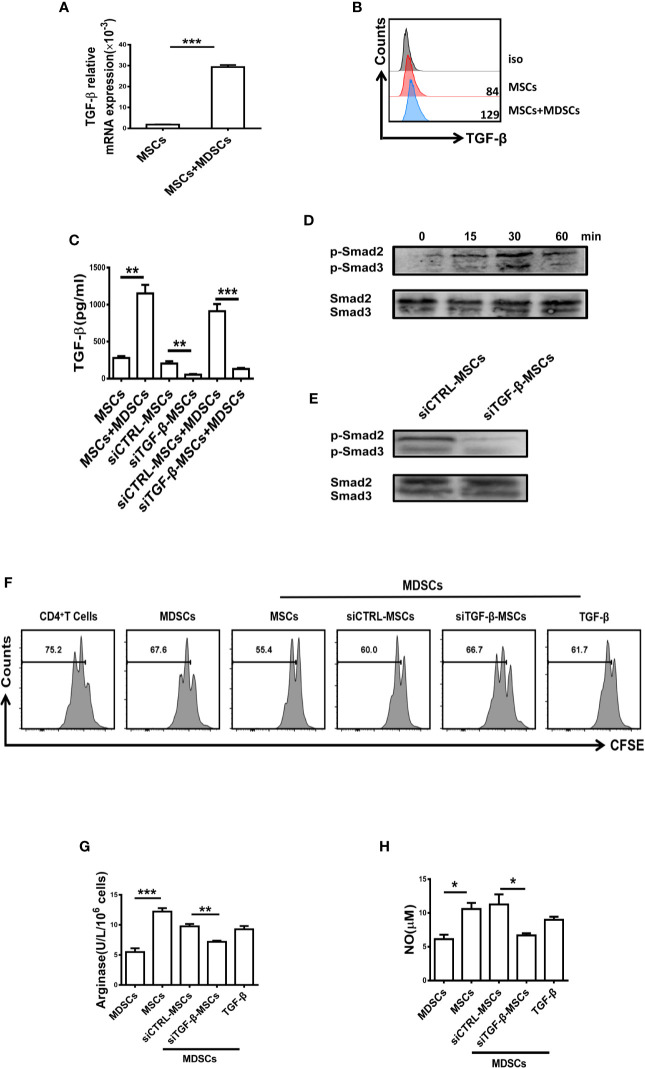
The suppressive capacity of MDSCs was enhanced by BM-MSCs-secreted TGF-β. **(A, B)** The mRNA level **(A)** and protein level **(B)** of TGF-β in BM-MSCs co-cultured with MDSCs were analyzed by qRT-PCR and flow cytometry respectively. **(C)** The concentration of TGF-β in the conditioned medium of control MSCs and TGF-β silenced BM-MSCs with or without MDSCs were measured by ELISA. **(D, E)** The expression of phosphorylated Smad2/3 in MDSCs co-cultured with MSCs or TGF-β-silenced MSCs was determined by western blot. **(F)** TGF-β-silenced MSCs were co-cultured with MDSCs for 48 h, then MDSCs were collected to co-culture with CD4^+^T cells in the presence of anti-CD3 and anti-CD28 mAbs for 72 h (MDSC:T cell ratio 1:1). CD4^+^ T cell proliferation was evaluated by staining with CFSE. Recombinant mouse TGF-β (0.8ng/ml) was used as a control. **(G, H)** The activity of arginase activity and the level of NO were detected in each group. The Data are shown as mean ± SD from three independent experiments. ***p < 0.001, **p < 0.01, *p < 0.05. NS, no significance.

It has been reported that TGF-β plays an important role in the inhibitory effect of MSCs (26, 27), and TGF-β has been shown to regulate the suppressive function of MDSCs (28). As shown in **Figures 4A, B**, a high level of TGF-β was measured in BM-MSCs when co-cultured with MDSCs. Similarly, the level of TGF-β in the supernatant of MDSCs co-cultured with MSCs was significantly enhanced. However, Co-culture of TGF-β-silenced MSCs and MDSCs showed strikingly reduced TGF-β in the supernatant (**Figure 4C**). Moreover, the phosphorylation of Smad2/3 in MDSCs was significantly increased after BM-MSCs treatment (**Figure 4D**), whereas knocking down the TGF-β led to the reduced activation of Smad2/3 (**Figure 4E**). To further clarify the critical role of TGF-β in regulating the suppressive effect of MDSCs, siRNA was used to silence TGF-β in BM-MSCs. After inhibition of TGF-β, the effect of BM-MSCs on the regulation of MDSCs was almost disappeared, MDSCs still exhibited low immunosuppressive function on T cell proliferation (**Figure 4F**), and the production of arginase and NO were also at low levels (**Figures 4G, H**). These *in vitro* data suggest that the enhanced suppressive effect of MDSCs was mainly mediated by TGF-β released by BM-MSCs.

### Silencing TGF-β in BM-MSCs Attenuates Their Capacity in Alleviating ESS Progression

**Figure 5:**
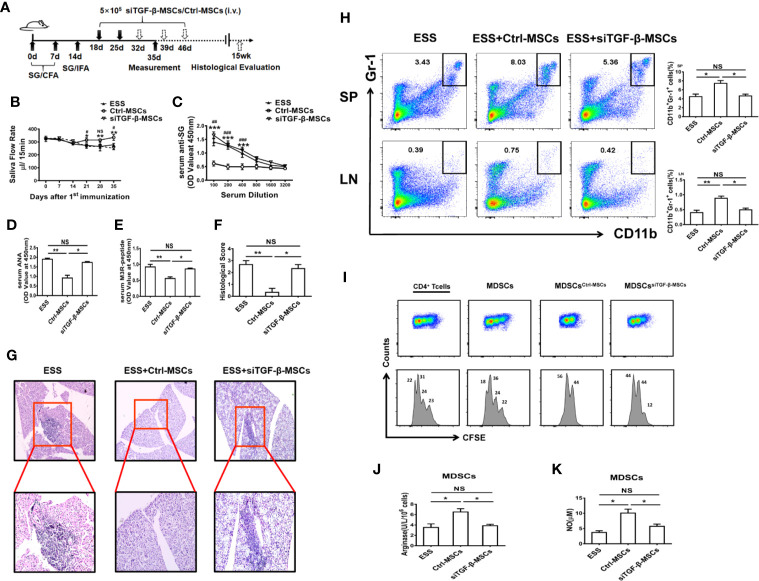
Knocking down TGF-β in BM-MSCs impairs their capability in inhibiting ESS development. **(A)** Graphic scheme of ESS induction and MSCs treatment. BM-MSCs transfected with TGF-β siRNA (siTGF-β) or negative control for 24 h, and then 5×10^5^ siTGF-β-MSCs or Ctrl-MSCs were intravenously injected on days 18 and 25 after the first immunization. Mice were sacrificed on day 35 (n=6). **(B)** The saliva flow rates were observed in each group. **(C–E)** Autoantibodies against SG antigens **(C)**, ANA **(D)**, and anti-M3R antibodies **(E)** were analyzed in the serum of mice with different treatment on day 35. **(F, G)** ESS mice were transferred with different BM-MSCs days 18, 25, 32, 39, and 46, the histological evaluation of glandular destruction in each group was performed on tissue sections of submandibular glands with H&E staining 15 weeks post first immunization. **(H)** Percentages of MDSCs in spleen and LNs were measured in each group on day 35. **(I)** MDSCs from different groups were isolated on day 35, and then co-cultured with CD4^+^T cells in the presence of anti-CD3 and anti-CD28 mAbs for 72 h (MDSC:T cell ratio 1:1). CD4^+^ T cell proliferation was evaluated by staining with CFSE. **(J, K)** The activity of arginase activity and the level of NO were detected in each group on day 35. Data are shown as mean± SD of three independent experiments, n=6/group. ***^/###^p < 0.001, **^/##^p < 0.01, *^/#^p < 0.05, NS, no significance. * represents Ctrl-MSCs *vs.* siTGF-β-MSCs, ^#^represents Ctrl-MSCs *vs.* ESS.

To further determine the role of TGF-β from BM-MSCs in regulating the function of MDSCs *in vivo*, we adoptively transferred BM-MSCs with silenced TGF-β expression into ESS mice (**Figure 5A**). As expected, the therapeutic effect of BM-MSCs in inhibiting ESS development was almost abolished after knocking down the TGF-β. Notably, the saliva flow rate was decreased, and the serum autoantibodies against total SG antigens, ANA, and anti-M3R Abs were remarkably increased in siTGFβ-MSCs-treated ESS mice (**Figures 5B–E**). In addition, the histological analysis showed serious lymphocytic infiltration in SG when compared to the Ctrl-MSCs-treated group (**Figures 5F, G**). Moreover, the percentages of MDSCs in spleen and LNs were significantly reduced in siTGFβ-MSCs-treated ESS mice (**Figure 5H**), and displayed weak suppressive capacity while the control group possessed strong suppression on T cells (**Figures 5I–K**). Taken together, these data suggest that BM-MSCs modulated the function of MDSCs is mainly mediated by TGF-β.

## Discussion

Extensive studies have described the role of MDSCs in the progression of various autoimmune diseases, including MS, RA, SLE, and type I diabetes (10–12, 14, 15, 29). Although there have been a large number of studies on MDSCs in autoimmune diseases, the exact effect of MDSCs in these diseases is still controversial. It has been found that adoptive transfer of MDSCs from EAE mice could obviously inhibit the inflammatory immune responses and suppress the progression of EAE (14). However, some other studies have shown that MDSCs in EAE could promote the differentiation of Th17 cells, and the severity of disease can be alleviated after depletion of MDSCs *in vivo* (15). The similar conflict results were also observed in RA. Some data showed the protective role of MDSCs during the development of collagen-induced arthritis (CIA) while some others found MDSCs significantly exacerbated the disease (10, 11). The controversial results on the role of MDSCs in autoimmune disorders are mainly due to the high heterogeneity and plasticity of MDSCs whose phenotypes and functions are largely dependent on the local microenvironment (9, 30, 31). MDSCs at different stages of disease may play a different role in either exacerbating or alleviating the disease. In our previous studies, we have investigated the role of MDSCs and their subsets in pSS. We found that MDSCs gradually lost their suppressive capacity during the development of ESS, thus leading to the progression of the disease. Early-stage MDSCs in ESS showed strong suppressive capacity while the late-stage MDSCs exhibited weak inhibitory effects on T cells. Therefore, reversing or enhancing the suppressive capacity of MDSCs *in vivo* might be a promising strategy for the treatment of SS. As expected, in this study, we found that BM-MSCs with immunomodulatory capacity could efficiently enhance the suppressive function of MDSCs, reserving the phenotype of MDSCs to an immature status with low levels of CD40, CD80, CD86, and MHC-II, and eventually alleviating the development of the disease.

Due to the strong immune regulatory property, MSCs have been applied in a number of autoimmune diseases. Indeed, the mechanisms for the immunomodulatory effect of MSCs has been extensively investigated. Abundant evidence has shown that MSCs exert modulatory effects on both innate and adaptive immune cells. MSCs can inhibit the activation, proliferation and differentiation of T cells (32–34). Krampera et al. demonstrated that murine BMSCs inhibited naive and memory T-cell responses to their cognate antigens (35). MSCs can also regulate the balance of Th1/Th2 cells, MSCs inhibit the production of IFN-γ by Th1 cells and increase the production of IL-4 by Th2 cells (36). Rafei et al. reported that MSCs could suppress Th17 cell activation in a CC chemokine ligand 2-dependent manner (34). Recently, MSCs are found to suppress Tfh cell differentiation in RA partially through the production of indoleamine 2,3-dioxygenase (IDO) (37), and the similar results are also observed in pSS patients and lupus-prone mice (38, 39). Besides, BM-MSCs can induce the differentiation of CD4^+^CD25^hi^Foxp3^+^ regulatory T cells and maintain their suppressive function (40). In addition to the regulation on T cells, Corcione et al. found that BM-MSC could inhibit the function, differentiation and the chemotactic properties of B cells (41). In relation to innate immune cells, MSCs have been demonstrated to inhibit the differentiation and function of DCs (42, 43). In pSS, it has been reported that MSCs can alleviate the disease by inhibiting Th1, Th17, and Tfh cell responses (38, 44). Moreover, MSCs can also ameliorate SS *via* suppressing IL-12 production in DCs (45). A recent study by Yao et al. has found that MSC-secreted interferon-β (IFN-β) promoted DCs to produce IL-27 and then suppressing the SS-like syndrome (46). In this study, we were the first to observe the regulation of MSCs on MDSCs. Our previous study has clarified the critical role of MDSCs during the progression of ESS, and the suppressive function of MDSCs was decreased during the disease development. We then found that BM-MSCs could directly modulate the suppressive function of MDSCs both *in vitro* and *in vivo*. After BM-MSCs treatment, the suppressive function of MDSCs was significantly enhanced with high levels of arginase and NO. Furthermore, the expression of CD40, CD80, CD86, and MHCII was also down-regulated. These data suggest that BM-MSCs can efficiently restore the strong suppressive function and the immature status of MDSCs and then alleviate the progression of ESS.

MSCs exhibited a range of immunomodulatory effect through releasing soluble factors and cell-cell contact. It has been reported that soluble factors, including TGF-β1, prostaglandin E2, indoleamine-pyrrole 2,3-dioxygenase, have been proposed to mediate the immunosuppressive function of MSCs (47). In this study, we observed high concentration of TGF-β in MSCs when co-cultured with MDSCs. TGF-β1 is a pleiotropic cytokine which has broad effects on the differentiation and function of various cell (48). Lee et al. have found Treg-derived TGF-β could efficiently promote MDSC proliferation and function in murine colitis (49). A recent study also reported that TGF-β could increase the expansion of MDSCs and enhance the suppressive capacity of MDSCs *in vitro* (28). Similarly, in our experiment, the canonical signaling of TGF-β was activated, the phosphorylation of Smad2 and Smad3 was strikingly enhanced in MDSCs co-cultured with BM-MSCs. Furthermore, while the TGF-β pathway in MDSCs was blocked by anti-TGF-β neutralizing antibody, the effect of BM-MSCs induced was almost inhibited, the suppressive function of MDSCs was reversed to the primary weak status. Concurrently, the *in vivo* experiment also showed TGF-β-silenced MSCs displayed a worse therapeutic effect in treating mice with ESS when compared with the control group. All these data indicate the regulation of BM-MSCs on the function of MDSCs was mainly mediated by TGF-β.

In conclusion, our findings suggest that BM-MSCs are capable of enhancing the suppressive capacity of MDSCs, thus alleviating the progression of ESS. Further exploration revealed the regulation of BM-MSCs on MDSCs was mainly through TGF-β/Smad pathway. Our study further enriches the mechanism of MSCs in the cell-based immunotherapy for autoimmune diseases.

## Data Availability Statement

The raw data supporting the conclusions of this article will be made available by the authors, without undue reservation.

## Ethics Statement

The animal study was reviewed and approved by Jiangsu University Animal Ethics and Experimentation Committee.

## Author Contributions

JT and YH performed the experiments, analyzed the data, and wrote the paper. QZ, HG, YueZ, and XA performed the experiments. HZ, YiZ, and ZS analyzed the data. SW, KR, and HX designed the study and wrote the paper. FZ revised the paper. All authors contributed to the article and approved the submitted version.

## Funding

This work was supported by the National Natural Science Foundation of China (Grant Nos. 81971542, 81701612), Natural Science Foundation of Jiangsu (Grant No. BK20170563), Project funded by China Postdoctoral Science Foundation (Grant No. 2017T100336), Summit of the Six Top Talents Program of Jiangsu Province (Grant No. 2017-YY-006), and Jiangsu Province’s Key Medical Talents Program (Grant No. ZDRCB2016018).

## Conflict of Interest

The authors declare that the research was conducted in the absence of any commercial or financial relationships that could be construed as a potential conflict of interest.

## References

[1] Fox RI Sjögren syndrome. Lancet (2005) 366(9482):321–31. 10.1016/S0140-6736(05)66990-5 16039337

[2] Fox PC Autoimmune diseases and Sjögren syndrome: an autoimmune exocrinopathy. Ann N Y Acad Sci (2007) 1098:15–21. 10.1196/annals.1384.003 17332090

[3] Nocturne G Mariette X Advances in understanding the pathogenesis of primary Sjögren syndrome. Nat Rev Rheumatol (2013) 9(9):544–56. 10.1038/nrrheum.2013.110 23857130

[4] Lin X Rui K Deng J Tian J Wang X Wang S Th17 cells play a critical role in the development of experimental Sjögren’s syndrome. Ann Rheum Dis (2015) 74(6):1302–10. 10.1136/annrheumdis-2013-204584 24573745

[5] Saito M Otsuka K Ushio A Yamada A Arakaki R Kudo Y Unique Phenotypes and Functions of Follicular Helper T Cell and Regulatory T Cell in Sjögren’s Syndrome. Curr Rheumatol Rev (2018) 14(3):239–45. 10.2174/1573397113666170125122858 PMC6225342 28124612

[6] Fu W Liu X Lin X Feng H Sun L Li S Deficiency in T follicular regulatory cells promotes autoimmunity. J Exp Med (2018) 215(3):815–25. 10.1084/jem.20170901 PMC5839755 29378778

[7] Tian J Rui K Hong Y Wang X Xiao F Lin X Increased GITRL Impairs the Function of Myeloid-Derived Suppressor Cells and Exacerbates Primary Sjögren Syndrome. J Immunol (2019) 202(6):1693–703. 10.4049/jimmunol.1801051 30760623

[8] Saraux A Pers JO Devauchelle-Pensec V Treatment of primary Sjögren syndrome. Nat Rev Rheumatol (2016) 12(8):456–71. 10.1038/nrrheum.2016.100 27411907

[9] Gabrilovich DI Nagaraj S Myeloid-derived suppressor cells as regulators of the immune system. Nat Rev Immunol (2009) 9(3):162–74. 10.1038/nri2506 PMC2828349 19197294

[10] Fujii W Ashihara E Hirai H Nagahara H Kajitani N Fujioka K Myeloid-derived suppressor cells play crucial roles in the regulation of mouse collagen-induced arthritis. J Immunol (2013) 191(3):1073–81. 10.4049/jimmunol.1203535 23804709

[11] Guo C Hu F Yi H Feng Z Li C Shi L Myeloid-derived suppressor cells have a proinflammatory role in the pathogenesis of autoimmune arthritis. Ann Rheum Dis (2016) 75(1):278–85. 10.1136/annrheumdis-2014-205508 PMC4418961 25371442

[12] Wu H Zhen Y Ma Z Li H Yu J Xu ZG Arginase-1-dependent promotion of TH17 differentiation and disease progression by MDSCs in systemic lupus erythematosus. Sci Transl Med (2016) 8(331):331ra40. 10.1126/scitranslmed.aae0482 PMC4895207 27009269

[13] Yin B Ma G Yen CY Zhou Z Wang GX Divino CM Myeloid-derived suppressor cells prevent type 1 diabetes in murine models. J Immunol (2010) 185(10):5828–34. 10.4049/jimmunol.0903636 PMC4355963 20956337

[14] Ioannou M Alissafi T Lazaridis I Deraos G Matsoukas J Gravanis A Crucial role of granulocytic myeloid-derived suppressor cells in the regulation of central nervous system autoimmune disease. J Immunol (2012) 188(3):1136–46. 10.4049/jimmunol.1101816 22210912

[15] Yi H Guo C Yu X Zuo D Wang XY Mouse CD11b+Gr-1+ myeloid cells can promote Th17 cell differentiation and experimental autoimmune encephalomyelitis. J Immunol (2012) 189(9):4295–304. 10.4049/jimmunol.1200086 PMC3478426 23034169

[16] Pittenger MF Mackay AM Beck SC Jaiswal RK Douglas R Mosca JD Multilineage potential of adult human mesenchymal stem cells. Science (1999) 284(5411):143–7. 10.1126/science.284.5411.143 10102814

[17] Wang Y Chen X Cao W Shi Y Plasticity of mesenchymal stem cells in immunomodulation: pathological and therapeutic implications. Nat Immunol (2014) 15(11):1009–16. 10.1038/ni.3002 25329189

[18] Nauta AJ Fibbe WE Immunomodulatory properties of mesenchymal stromal cells. Blood (2007) 110(10):3499–506. 10.1182/blood-2007-02-069716 17664353

[19] Sun L Wang D Liang J Zhang H Feng X Wang H Umbilical cord mesenchymal stem cell transplantation in severe and refractory systemic lupus erythematosus. Arthritis Rheum (2010) 62(8):2467–75. 10.1002/art.27548 20506343

[20] Gu Z Akiyama K Ma X Zhang H Feng X Yao G Transplantation of umbilical cord mesenchymal stem cells alleviates lupus nephritis in MRL/lpr mice. Lupus (2010) 19(13):1502–14. 10.1177/0961203310373782 20647254

[21] Augello A Tasso R Negrini SM Cancedda R Pennesi G Cell therapy using allogeneic bone marrow mesenchymal stem cells prevents tissue damage in collagen-induced arthritis. Arthritis Rheum (2007) 56(4):1175–86. 10.1002/art.22511 17393437

[22] Liang J Zhang H Wang D Feng X Wang H Hua B Allogeneic mesenchymal stem cell transplantation in seven patients with refractory inflammatory bowel disease. Gut (2012) 61(3):468–9. 10.1136/gutjnl-2011-300083 21617158

[23] Christopeit M Schendel M Foll J Muller LP Keysser G Behre G Marked improvement of severe progressive systemic sclerosis after transplantation of mesenchymal stem cells from an allogeneic haploidentical-related donor mediated by ligation of CD137L. Leukemia (2008) 22(5):1062–4. 10.1038/sj.leu.2404996 17972956

[24] Scardina GA Spano G Carini F Spicola M Valenza V Messina P Diagnostic evaluation of serial sections of labial salivary gland biopsies in Sjögren syndrome. Med Oral Patol Oral Cir Bucal (2007) 12(8):E565–8. 18059240

[25] Tian J Rui K Tang X Ma J Wang Y Tian X MicroRNA-9 Regulates the Differentiation and Function of Myeloid-Derived Suppressor Cells via Targeting Runx1. J Immunol (2015) 195(3):1301–11. 10.4049/jimmunol.1500209 26091714

[26] Dazzi F Lopes L Weng L Mesenchymal stromal cells: a key player in ‘innate tolerance’? Immunology (2012) 137(3):206–13. 10.1111/j.1365-2567.2012.03621.x PMC3482678 22804624

[27] Patel SA Meyer JR Greco SJ Corcoran KE Bryan M Rameshwar P Mesenchymal stem cells protect breast cancer cells through regulatory T cells: role of mesenchymal stem cell-derived TGF-beta. J Immunol (2010) 184(10):5885–94. 10.4049/jimmunol.0903143 20382885

[28] Lee CR Lee W Cho SK Park SG Characterization of Multiple Cytokine Combinations and TGF-beta on Differentiation and Functions of Myeloid-Derived Suppressor Cells. Int J Mol Sci (2018) 19(3):869. 10.3390/ijms19030869 PMC5877730 29543758

[29] Ji J Xu J Zhao S Liu F Qi J Song Y Myeloid-derived suppressor cells contribute to systemic lupus erythaematosus by regulating differentiation of Th17 cells and Tregs. Clin Sci (Lond) (2016) 130(16):1453–67. 10.1042/CS20160311 27231253

[30] Cripps JG Gorham JD MDSC in autoimmunity. Int Immunopharmacol (2011) 11(7):789–93. 10.1016/j.intimp.2011.01.026 PMC3109222 21310255

[31] Melero-Jerez C Ortega MC Moline-Velazquez V Clemente D Myeloid derived suppressor cells in inflammatory conditions of the central nervous system. Biochim Biophys Acta (2016) 1862(3):368–80. 10.1016/j.bbadis.2015.10.015 26527182

[32] Chen M Su W Lin X Guo Z Wang J Zhang Q Adoptive transfer of human gingiva-derived mesenchymal stem cells ameliorates collagen-induced arthritis via suppression of Th1 and Th17 cells and enhancement of regulatory T cell differentiation. Arthritis Rheum (2013) 65(5):1181–93. 10.1002/art.37894 PMC4364405 23400582

[33] Gonzalez-Rey E Gonzalez MA Varela N O’Valle F Hernandez-Cortes P Rico L Human adipose-derived mesenchymal stem cells reduce inflammatory and T cell responses and induce regulatory T cells in vitro in rheumatoid arthritis. Ann Rheum Dis (2010) 69(1):241–8. 10.1136/ard.2008.101881 19124525

[34] Rafei M Campeau PM Aguilar-Mahecha A Buchanan M Williams P Birman E Mesenchymal stromal cells ameliorate experimental autoimmune encephalomyelitis by inhibiting CD4 Th17 T cells in a CC chemokine ligand 2-dependent manner. J Immunol (2009) 182(10):5994–6002. 10.4049/jimmunol.0803962 19414750

[35] Krampera M Glennie S Dyson J Scott D Laylor R Simpson E Bone marrow mesenchymal stem cells inhibit the response of naive and memory antigen-specific T cells to their cognate peptide. Blood (2003) 101(9):3722–9. 10.1182/blood-2002-07-2104 12506037

[36] Aggarwal S Pittenger MF Human mesenchymal stem cells modulate allogeneic immune cell responses. Blood (2005) 105(4):1815–22. 10.1182/blood-2004-04-1559 15494428

[37] Liu R Li X Zhang Z Zhou M Sun Y Su D Allogeneic mesenchymal stem cells inhibited T follicular helper cell generation in rheumatoid arthritis. Sci Rep (2015) 5:12777. 10.1038/srep12777 26259824 PMC4531289

[38] Liu R Su D Zhou M Feng X Li X Sun L Umbilical cord mesenchymal stem cells inhibit the differentiation of circulating T follicular helper cells in patients with primary Sjögren syndrome through the secretion of indoleamine 2,3-dioxygenase. Rheumatol (Oxford) (2015) 54(2):332–42. 10.1093/rheumatology/keu316 25169988

[39] Yang X Yang J Li X Ma W Zou H Bone marrow-derived mesenchymal stem cells inhibit T follicular helper cell in lupus-prone mice. Lupus (2018) 27(1):49–59. 10.1177/0961203317711013 28537524

[40] Selmani Z Naji A Zidi I Favier B Gaiffe E Obert L Human leukocyte antigen-G5 secretion by human mesenchymal stem cells is required to suppress T lymphocyte and natural killer function and to induce CD4+CD25highFOXP3+ regulatory T cells. Stem Cells (2008) 26(1):212–22. 10.1634/stemcells.2007-0554 17932417

[41] Corcione A Benvenuto F Ferretti E Giunti D Cappiello V Cazzanti F Human mesenchymal stem cells modulate B-cell functions. Blood (2006) 107(1):367–72. 10.1182/blood-2005-07-2657 16141348

[42] Jiang XX Zhang Y Liu B Zhang SX Wu Y Yu XD Human mesenchymal stem cells inhibit differentiation and function of monocyte-derived dendritic cells. Blood (2005) 105(10):4120–6. 10.1182/blood-2004-02-0586 15692068

[43] Djouad F Charbonnier LM Bouffi C Louis-Plence P Bony C Apparailly F Mesenchymal stem cells inhibit the differentiation of dendritic cells through an interleukin-6-dependent mechanism. Stem Cells (2007) 25(8):2025–32. 10.1634/stemcells.2006-0548 17510220

[44] Xu J Wang D Liu D Fan Z Zhang H Liu O Allogeneic mesenchymal stem cell treatment alleviates experimental and clinical Sjögren syndrome. Blood (2012) 120(15):3142–51. 10.1182/blood-2011-11-391144 PMC3471521 22927248

[45] Shi B Qi J Yao G Feng R Zhang Z Wang D Mesenchymal stem cell transplantation ameliorates Sjögren syndrome via suppressing IL-12 production by dendritic cells. Stem Cell Res Ther (2018) 9(1):308. 10.1186/s13287-018-1023-x 30409219 PMC6225717

[46] Yao G Qi J Liang J Shi B Chen W Li W Mesenchymal stem cell transplantation alleviates experimental Sjögren syndrome through IFN-beta/IL-27 signaling axis. Theranostics (2019) 9(26):8253–65. 10.7150/thno.37351 PMC6857067 31754394

[47] Uccelli A Moretta L Pistoia V Mesenchymal stem cells in health and disease. Nat Rev Immunol (2008) 8(9):726–36. 10.1038/nri2395 19172693

[48] Kumar V Patel S Tcyganov E Gabrilovich DI The Nature of Myeloid-Derived Suppressor Cells in the Tumor Microenvironment. Trends Immunol (2016) 37(3):208–20. 10.1016/j.it.2016.01.004 PMC4775398 26858199

[49] Lee CR Kwak Y Yang T Han JH Park SH Ye MB Myeloid-Derived Suppressor Cells Are Controlled by Regulatory T Cells via TGF-beta during Murine Colitis. Cell Rep (2016) 17(12):3219–32. 10.1016/j.celrep.2016.11.062 28009291

